# Immune checkpoint pathways in immunotherapy for head and neck squamous cell carcinoma

**DOI:** 10.1038/s41368-020-0084-8

**Published:** 2020-05-28

**Authors:** Zi Mei, Junwen Huang, Bin Qiao, Alfred King-yin Lam

**Affiliations:** 1 Department of Oral and Maxillofacial Surgery, The First Affiliated Hospital of Zhengzhou University, Zhengzhou, China; 2 Cancer Molecular Pathology and Griffith Medical School, Griffith University, Gold Coast, Queensland Australia

**Keywords:** Oral cancer, Cancer immunotherapy

## Abstract

With the understanding of the complex interaction between the tumour microenvironment and immunotherapy, there is increasing interest in the role of immune regulators in the treatment of head and neck squamous cell carcinoma (HNSCC). Activation of T cells and immune checkpoint molecules is important for the immune response to cancers. Immune checkpoint molecules include cytotoxic T lymphocyte antigen 4 (CTLA-4), programmed death 1 (PD-1), T-cell immunoglobulin mucin protein 3 (TIM-3), lymphocyte activation gene 3 (LAG-3), T cell immunoglobin and immunoreceptor tyrosine-based inhibitory motif (TIGIT), glucocorticoid-induced tumour necrosis factor receptor (GITR) and V-domain Ig suppressor of T cell activation (VISTA). Many clinical trials using checkpoint inhibitors, as both monotherapies and combination therapies, have been initiated targeting these immune checkpoint molecules. This review summarizes the functional mechanism and use of various immune checkpoint molecules in HNSCC, including monotherapies and combination therapies, and provides better treatment options for patients with HNSCC.

## Introduction

According to the Global Cancer Report in 2018, head and neck squamous cell carcinoma (HNSCC) was the eighth most frequent cancer in the world, and the mortality rate ranked eighth among all cancers.^1^ Despite the trend of improved survival rates for patients with cancer over the past 20 years, local and distant failure after treatment of advanced HNSCC occur in up to 40% and 30% of patients, respectively.^2^

The past two decades have seen a surge in medical research surrounding cancer treatments, resulting in new strategies that have provided hope that we may effectively treat many cancers. We have improved our understanding of the pathology and molecular details of cancer and can perform gene sequencing based on individual cancer cells to obtain more “personalized treatment”. Clinical trials of gene-targeting agents have successfully laid the foundation for other cancer therapies.

**Fig. 1:**
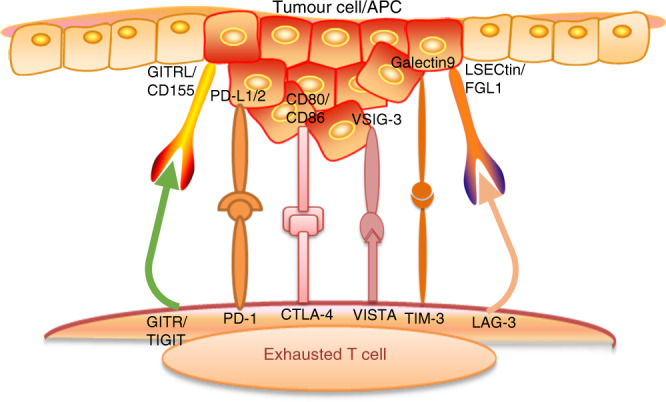
The coinhibitory pathways of head and neck squamous cell carcinoma. During carcinogenesis, T cell activation is inhibited by a number of pathways, which are often activated by the expression of certain ligands on tumour cells or antigen-presenting cells (PD-L1/2, CD80/CD86, galectin 9, LSECtin/FGL1, CD155/GITRL, and VSIG-3) that bind to receptors on T cells (PD-1, CTLA-4, TIM-3, LAG-3, TIGIT, GITR, and VISTA) and inhibit the activation and the anti-tumour function of T cells

With the discovery of cancer immunotherapy and a deeper understanding of the T cell responses to targeted immune checkpoint therapies, as well as the success of clinical trials of drugs that block immunological checkpoints, research predicting and identifying precise biomarkers of genomically targeted agents will become more popular. Because the immune response is dynamic and variable, identifying a useful drug in the field of cancer immunotherapy may involve more than just identifying a single biomarker to select a subset of patients for treatment. Therefore, we must evaluate the timeliness of the immune response and determine the effect of the immune response on the clinical treatment to achieve the best therapeutic effect with combination treatments (Fig. 1).

HNSCC tissues are in an immunosuppressive state, with fewer lymphocytes, natural killer (NK) cells and specific antigens than normal tissues.^3^ Thus, this cancer type is called a “cold tumour”. HNSCC can escape immune surveillance through different mechanisms.

Immunological checkpoint inhibitors, which target T cell regulatory pathways to enhance antitumour immune efficiency, have brought great clinical advances and offer new methods for anticancer treatment. In addition to surgery, radiotherapy and chemotherapy, targeting immune checkpoints has become another good choice for clinicians to treat HNSCC. To date, the U.S. Food and Drug Administration (FDA) has approved many immune checkpoint drugs for clinical usage.

### Tumour microenvironment of HNSCC

The immune system plays a key role in the formation, development, and progression of HNSCC. There are more infiltrating regulatory T cells (Tregs) in patients with human papillomavirus-positive (HPV^+^) HNSCC than in those with HPV^-^ HNSCC. Studies have shown that high levels of cluster of differentiation (CD) 8^+^ tumour-infiltrating lymphocytes (TILs) in HPV^+^ HNSCC tissues are associated with improved disease-free survival.^4^ Primed effector CD8^+^ cytotoxic T lymphocytes (CTLs) can exit the lymphoid tissue, move towards their target antigens, and identify such antigens in the context of major histocompatibility complex (MHC) class I molecules for target cell lysis. In addition, the number of CD4^+^ T helper (Th1) lymphocytes has a significant correlation with CD8^+^ T cell density in the tumour microenvironment.^5^ In peripheral blood, T helper 17 (Th17) and T cytotoxic 17 (Tc17) cells were significant predictors of poor prognosis for patients with HNSCC.^6^

### Mechanism of immune escape in HNSCC

Immunological checkpoints are immunosuppressive molecules that can maintain self-tolerance by modulating T cell function and protecting surrounding tissues by suppressing immune responses. Tumour cells can take advantage of this feature to evade attack by immune cells. In patients with HNSCC, immune checkpoints are the basic mechanism leading to cancer cell escape. To eliminate HNSCC using immunotherapy, specific identification of tumour cells and lysis by CTLs are required. The human lymphocyte antigen (HLA) system is complex and polymorphic, and is the human version of the major histocompatibility complex (MHC) system. HLA molecules are mainly present on the surface of antigen-presenting cells (APCs) and present tumour antigen peptides to CTLs.^7^ By altering the expression of HLA-I and impairing antigen processing machinery (APM), HNSCC cells can reduce tumour antigen recognition by T cells. Through the sequencing of HNSCC cells, multiple mutations have been detected in HLA alleles and antigen processing machinery components. Chromosome and regulatory defects related to genes encoding HLA and APM factors can lead to the selective deletion of HLA and APM components in a large number of HNSCCs and are associated with a worse prognosis of patients.^8^

Tumour cells with complete loss of HLA can escape the immune response by evading recognition by T cells. However, this can trigger the activation of NK cells because the loss of HLA can eliminate suppression of NK cell activation.^9^ Therefore, tumour cells can use a variety of mechanisms to achieve immune evasion while avoiding complete loss of HLA expression.^10^

In summary, downregulation or deletion of HLA class I molecules or APM components is one of the strategies by which tumours evade the immune response. In addition, tumour cells secrete immunosuppressive and pro-apoptotic factors, as well as upregulate inhibitory cell surface molecules that can lead to evasion of the immune response.

## The basic biology of immune checkpoints

### Activation and regulation of T cells

T cell activation requires dual signalling: the first signal is induced when the T cell receptor (TCR) recognizes the MHC-antigen; the second signal is mediated by the B7 molecule on the surface of the APC and the CD28 molecule on the surface of the T cell, which are also known as co-stimulatory factors. Without either of the two signals, T cells cannot be activated. Importantly, the initiation and activation of T cells are regulated by central and peripheral checkpoints.^11^ Activation of T cells is bidirectional; in addition to initiating proliferation and differentiation, T cell activation also induces an inhibitory pathway that ultimately attenuates and terminates T cell responses.

### Function of immune checkpoints in HNSCC

An immune checkpoint is a regulatory signal that affects immune activation and self-tolerance. Immune checkpoint signalling is critical for preventing autoimmunity and protecting host tissues from immune-mediated indirect damage (Fig. 1).

#### Cytotoxic T lymphocyte antigen 4 (CTLA-4)

As the first clinically targeted immune checkpoint receptor, CTLA-4 is expressed primarily in T cells and is less expressed in active B cells, monocytes, granulocytes and dendritic cells (DCs).^12^ CTLA-4 is also expressed on Tregs and produces the immunosuppressive molecule transforming growth factor-β (TGF-β) when activated with CD28.^13^ Both CTLA-4 and CD28 act as transmembrane receptors. CTLA-4 can bind the B7 protein to induce T cell dysfunction and participate in negative regulation of the immune response. Under normal circumstances, the immunosuppressive effect of CTLA-4 is to stimulate the immune response effectively without excessive damage to normal tissues. However, cancer cells secrete TGF-β, which can stimulate the expression of CTLA-4, leading to T cell exhaustion.^14^ T cell exhaustion is a state in which T cells have weak functions and might exert immunosuppression. The affinity of CD28 for CTLA-4 on the surface of T cells exceeds its affinity for the co-stimulatory molecules CD80 and CD86. Thus, T cells are prevented from proliferating and fail to function.^15^

Both CTLA-4 and CD28 bind to the two ligands CD80 and CD86, which are expressed on the surface of antigen-presenting cells. In addition, activation of T cells also leads to the expression of CTLA-4, which aggregates in T cells at the T cell-antigen-presenting cell (APC) interface, reaching a level that ultimately prevents co-stimulatory stimulation and abolishes activated T cell responses. In addition, CTLA-4 is also expressed on Tregs. CTLA-4 is contains properties that cause it to sometimes localize to plasma membranes. Approximately 90% of CTLA-4 is intracellular. CTLA-4 also differs from CD28 in that it causes a high degree of endocytosis, with more than 80% of surface molecules being internalized within 5 min. In addition, researchers found that CTLA-4 could be released from the endosomes once internalized, and then the endosomes could be recycled to the plasma membrane. We observed limited colocalization of CTLA-4 with lysosomes in labelling experiments, yet other experiments have shown that CTLA-4 degradation can be inhibited by lysosomal blockade.^16^

#### Programmed cell death protein-1/programmed death-ligand 1 (PD-1/PD-L1)

PD-1 belongs to the CD28 receptor family and is mainly expressed on activated T cells and B cells. It is also found in monocytes and a small fraction of thymocytes. The ligands for PD-1 include PD-L1 and PD-L2. Both ligands are expressed on endothelial and epithelial antigen-presenting cells and activated lymphocytes.^17^ Although PD-L1 has limited expression in normal tissues, it is inducible and constitutively expressed in many solid and haematological malignancies. PD-L1 overexpression in tumour cells can promote tumour formation.^18^ In non-small-cell lung carcinoma (NSCLC) and melanoma, high expression of PD-L1 on tumour cells is strongly associated with high tumour grade and poor prognosis of patients.^19,20^ HNSCC tissues produce PD-L1 through an abnormal PD-1 signalling pathway, which leads to tumour immunosuppression.^21^ The PD-1/PD-L1 signalling pathway can be activated in a chronic inflammatory environment. In fact, the formation of HPV^+^ HNSCC is mainly due to the activation assisted by the PD-1/PD-L1 axis. HPV^+^ HNSCC tissues had more lymphocytes and higher levels of PD-L1 than HPV^-^ HNSCC tissues, and the infiltrated CTLs expressed more PD-1.^22^ PD-L1 can deliver additional immunosuppressive signals by binding to the CD80 receptor, which is expressed on T cells. Thus, depending on the immune environment within different HNSCC tumour tissues, the PD-1/PD-L1 axis can be blocked at different levels: targeting PD-1 can block its binding to PD-L1/PD-L2, whereas targeting PD-L1 can inhibit its binding to PD-1/CD80.

Interactions between PD-L1 and PD-1 can directly regulate the tumour microenvironment and have different functional significance in modulating the effects of T cells, DCs, bone marrow-derived suppressor cells (MDSCs) and Tregs. Blocking PD-1 on Tregs inhibits their ability to mediate immune tolerance, but this does not indicate that PD-1 can directly enhance Treg function.^23^ On effector T cells, PD-1 expression is considered an exhausted marker, and it also increases sensitivity to the PD-L1 death signalling pathway. The PD-L1 signalling pathway not only downregulates antitumour T cell function but also affects the cellular interactions between the innate immune response and the adaptive immune response, such as interactions between DCs, MDSCs and Tregs.

Because PD-1 and CTLA-4 belong to the same protein family and have similar structural fragments, Freeman and his colleagues speculated that PD-L1 is part of the B7 family.^24^ Inflammatory cytokines can induce PD-L1 expression, for example, type I and type II interferon (IFN), tumour necrosis factor-alpha (TNF-α) and vascular endothelial growth factor (VEGF). The PD-L2 molecule is only expressed on activated macrophages and dendritic cells. Once PD-L1 binds to PD-1, it will inhibit T cell receptor-mediated lymphocyte proliferation and cytokine secretion. In activated T cells, PD-1 binds to PD-L1 and then promotes phosphorylation of tyrosine in the immunoreceptor tyrosine-based switch motif (ITSM) domain of PD-1, which leads to dephosphorylation of downstream protein kinases, spleen tyrosine kinase (Syk) and phosphatidylinositol 3-kinase (PI3K), and inhibition of downstream protein kinase B (AKT) and extracellular regulated protein kinase (ERK). The activation of ion channels ultimately inhibits the transcription and translation of genes and cytokines required for T cell activation and plays a role in negatively regulating T cell activity.

#### T cell immunoglobulin mucin-3 (TIM-3)

As a member of the TIM family, TIM-3 was first reported in 2002 and was specifically expressed on CD4^+^ Th1 but not Th2 lymphocytes.^25^ It is also expressed on Tregs, DCs, monocytes, mast cells, NK cells and TILs. TIM-3 is also found on tumour cells, such as melanoma and B cell lymphoma cells.^26^ Studies have shown that TIM-3 and its ligands can regulate T cell tolerance. Galectin-9 has been identified as a major ligand for TIM-3 and is a member of the galectin family.^27^ It can regulate diverse biological functions of tumour cells, aggregation, adhesion and apoptosis.^28^ When TIM-3 binds to its ligand galectin-9, it can inhibit the expansion of Th1 and Th17 cells. This change will promote apoptosis of Th1 cells, deplete the function of CD8^+^ T cells, induce massive expansion of MDSCs and hamper the immune response. In the early stage of tumour formation, TIM-3^+^CD4^+^ T cells can secrete IFN-γ to have antitumor effects. On the other hand, in the middle and late stages of tumour formation, TIM-3^+^ Tregs proliferate, thereby blocking the function of effector T cells.^29^ When TIM-3 was blocked with an anti-TIM-3 monoclonal antibody, the antitumor response of T cells mediated by IFN-γ was promoted.^29^

The expression of TIM-3 is positively correlated with the survival of pancreatic ductal adenocarcinoma and renal cell carcinoma.^30,31^ However, The Cancer Genome Atlas (TCGA) database showed that the expression of TIM-3 is not associated with patient pathological tumour node metastasis (TNM) stage in HNSCC. On the other hand, TIM-3 expression has a positive relationship with the presence of lymph node metastasis and recurrence.^32^ Elevated expression of TIM-3 contributes to the exhaustion of effector T cells, which may lead to ineffective antitumour immune response and tumour clearance, resulting in cancer metastasis and recurrence in patients with HNSCC.^33^ When treatment with anti-TIM-3 antibodies was used in an HNSCC mouse model, the CD4^+^TIM-3^+^ to CD8^+^TIM-3^+^ ratio was affected, which led to an increase in the function of CD4^+^ and CD8^+^ T cells and a decrease in marrow-derived suppressor cells, significantly inhibiting tumorigenesis and improving antitumour immune responses.^32^

In autoimmune diseases, the expression of TIM-3 is increased in macrophages. M2 macrophages are alternatively activated (in contrast to M1 macrophages) by exposure to certain cytokines. Increased expression of TIM-3 in M2 macrophages participates in immune regulation by inhibiting macrophage activation.^31^ Another study also showed that upregulation of TIM-3 expression in mouse M2 macrophages could mediate inflammatory responses.^34^

#### Lymphocyte activated gene-3 (LAG-3)

The surface molecule LAG-3/CD223, a member of the immunoglobulin superfamily, was first discovered in 1990.^35^ It is mainly expressed on activated T cells and can be expressed on NK cells, B cells and plasmacytoid dendritic cells. LAG-3 negatively modulates T cell proliferation, activation and homeostasis^36^ and has similar functions to CTLA-4 and PD-1.^37^ LAG-3 plays a vital role in the inhibitory function of Tregs.^38^ In some malignancies, the coexpression of LAG-3 and PD-1 on tumour-infiltrating lymphocytes is related to the impaired function of CD8^+^ effector T cells, which promotes the immune escape of tumours.^39^ LAG-3 has a similar protein sequence to the CD4 receptor. One of its ligands is an MHC-II molecule, and LAG-3 has an even stronger affinity for this ligand than CD4.^40^ In fact, the effect of LAG-3 on CD8^+^ T cell function does not depend on the interaction with MHC-II; liver and lymph node sinusoidal endothelial cell C-type lectin (LSECtin) of the DC signalling family is considered to be another ligand for LAG-3. LSECtin binds to LAG-3 to inhibit the secretion of IFN-γ by effector T cells in melanoma, thereby inhibiting the antitumour immune response.^41^ Blocking LAG-3 leads to increased accumulation and effector function of CD8^+^ T cells in ovarian cancer.^42,43^

LAG-3, generally expressed on Tregs, is required for Treg cell-mediated T cell homeostasis. Blocking LAG-3 can inhibit Treg activation and abolish Treg inhibition, while the ectopic expression of LAG-3 by non-Treg CD4^+^ T cells contributes to inhibitory activity. LAG-3 is further expressed on CD4^+^Foxp3^+^, interleukin-10 (IL-10)-secreting type 1 regulated (Tr1) T cells. LAG-3 also has high expression on Tregs in HNSCC.^44^

In 2019, Wang and his colleagues found that fibrinogen-like protein 1 (FGL1), a liver-secreted protein, is a new ligand for LAG-3.^45^ FGL1 is the main LAG-3 functional ligand that functions independently of MHC-II. FGL1 inhibited the activation of antigen-specific T cells, and the ablation of mouse FGL1 promoted T cell immunity. Under normal physiological conditions, FGL1 is mainly secreted by liver cells and is involved in mitosis and metabolism.^46^ Blocking the FGL1-LAG-3 interaction with gene ablation or monoclonal antibodies enhances the T cell response and promotes antitumour immunity. FGL1 is upregulated in most human cancers and is associated with poor prognosis and treatment outcomes. The FGL1/LAG-3 pathway can be used as a potential target for immune escape mechanisms and cancer immunotherapy.

#### T cell immunoglobulin and immunoreceptor tyrosine-based inhibitory motif (TIGIT)

TIGIT is a member of the poliovirus receptor (PVR)/Nectin family, and is also known as Washington University cell adhesion molecule (WUCAM), V-set and transmembrane domain-containing 3 (Vstm3), V-set and immunoglobulin domain-containing 9 (VSIG9). It is mainly composed of four parts, namely, the extracellular immunoglobulin variable region (IgV) domain, a type 1 transmembrane domain, a classical ITIM and an immunoglobulin tyrosine tail (ITT) motif. TIGIT is expressed in lymphocytes, particularly in effector and regulatory CD4^+^ T cells, follicular helper CD4^+^ T cells, effective CD8^+^ T cells and NK cells.^47^ CD155 (PVR, Necl5 or Tage4) is a high-affinity ligand of TIGIT. It is also a cell surface receptor that is highly expressed on endothelial cells, fibroblasts, DCs and tumour cells.^48^ CD155 is highly expressed on the surface of tumours, binds to TIGIT on the surface of NK and T cells, increases IL-10 secretion, reduces the secretion of pro-inflammatory cytokines, and inhibits the antitumour immune response. CD112 (PVRL2 or nectin 2) and CD113 (PVRL3) also bind with TIGIT, but their affinity for TIGIT is weaker than that of CD155.

CD155, CD112, and CD113 share a ligand with CD226 (DNAM-1). The inhibition of TIGIT can counteract the activation of the co-stimulatory molecule CD226, and TIGIT/CD226 forms a network that regulates human T cell function, such as the CD28/CTLA-4-CD80/CD86 pathway. Coactivation receptors are expressed on naive and resting T cells, and corepression receptors are expressed after T cell activation. CD226 transmits a positive signal, and TIGIT transmits an inhibitory signal.^49^

#### Glucocorticoid induced TNFR family related gene (GITR)

GITR is a new addition to the tumour necrosis factor receptor (TNFR) superfamily and is expressed on the surface of CD25^+^CD4^+^ Tregs, effector T cells, and natural killer cells.^50^ Binding of GITR and its ligand GITRL can reduce the recruitment of Tregs, weaken their inhibitory function and activate the MAPK (mitogen-activated protein kinase)/ERK pathway and NF-κB signalling, increasing T cell proliferation, promoting secretion of pro-inflammatory cytokines, and enhancing antitumour function.^51,52^ Therefore, GITR is an immune checkpoint that can potentially be monitored after immunotherapy with an anti-GITR antibody (clone DTA-1) that blocks the inhibition of regulatory T cells.^53^

#### V-domain Ig suppressor of T cell activation (VISTA)

A newer checkpoint molecule, VISTA, also named differentiation of embryonic stem cells 1 (Dies1), Gi24, and PD-1 homologue (PD-1H), is similar in function to PD-L1 and can effectively inhibit T cell activation. In mice, VISTA is highly expressed on tumour-infiltrating leukocytes, while blockade enhances antitumour immunity in multiple tumour models.^54^ VISTA is mainly expressed on myeloid APCs and T cells, especially on Tregs.^55^ VISTA helps created an immunosuppressed tumour microenvironment by enhancing Treg maturation and inhibiting T cell activation. VISTA and PD-1 inhibit T cells through a non-redundant immune regulatory network and comodulate T cell responses.^56^

VSIG3 (V-set and immunoglobulin domain-containing 3) is a novel ligand for VISTA,^57^ also known as immunoglobulin superfamily member 11 (IGSF11) and brain and testicular-specific immunoglobulin superfamily 11 (BT-IgSF), that mediates homogenic adhesion in a calcium-independent manner.^58^ The interaction of VSIG3 and VISTA on activated T cells inhibits T cell proliferation and the production of cytokines and chemokines. The suppressive effect of VSIG3 on activated T cells and the high expression of VSIG3 in colorectal adenocarcinoma, hepatocellular carcinoma, and intestinal-type gastric carcinoma suggest that blocking the VSIG3/VISTA pathway could be a new cancer immunotherapy strategy.^57^

## Clinical trials of immune checkpoint-blocking therapeutics in HNSCC

### PD-1/PD-L1

Currently, monoclonal antibodies against humanized PD-1 and PD-L1 are being used in clinical trials for patients with advanced solid tumours under the approval of the FDA.^59^ Among them, immune checkpoint inhibitors targeting the PD-1/PD-L1 axis have been approved for clinical usage in some malignancies, including non-small-cell lung carcinoma, urothelial carcinoma, renal cell carcinoma, Merkle cell carcinoma and hepatocellular carcinoma.^60^ There are four approved monoclonal antibodies targeting PD-1 and PD-L1, namely, nivolumab (opdivo), pembrolizumab (keytruda), avelumab (bavencio) and atezolizumab (tecentriq). The first two are anti-PD-1 antibodies, and the last two are anti-PD-L1 antibodies. Nivolumab and pembrolizumab have been approved by the FDA for patients with HNSCC with relapse or metastasis who are cisplatin-resistant.^61^

Nivolumab is an anti-PD-1 monoclonal IgG4 antibody that blocks co-suppression signals via the PD-1/PD-L1 axis. It became the first immunotherapy approved by the FDA for patients with HNSCC based on a phase III clinical trial (Checkmate 141, NCT02105636) on November 10, 2016.^62^ The study randomly selected 361 patients with recurrent HNSCC who progressed six months after platinum chemotherapy. The patients were allocated at a 2:1 ratio to receive either nivolumab (at a dose of 3 mg·kg^−1^ of body weight) or standard single-drug systemic therapy every two weeks, such as methotrexate, docetaxel or cetuximab. The primary monitoring point was overall survival (OS) but objective response, progression-free survival (PFS), safety and patient-reported quality of life were also analysed.^63^ The results showed that the median survival of the group treated with nivolumab was 7.5 months, while the survival of the standard treatment group was 5.1 months (95% confidence interval [CI], *P* = 0.01). The estimated 1-year survival rates of the nivolumab and standard treatment groups were approximately 36.0% and 16.6%, respectively. The PFS rate of the nivolumab grpi[at 6 months was 19.7%, compared with 9.9% for the standard treatment group. In addition, the nivolumab group had fewer severe toxic effects (grade III or IV) than the standard treatment group (13.1% vs 35.1%).

Patients treated with nivolumab appeared to have longer OS than patients receiving standard treatment, regardless of the expression levels of PD-L1 or the p16 status of the cancer. There was no significant relationship between the levels of PD-L1 (high versus low) and p16 (positive versus negative) in tumours regardless of treatment type.

Pembrolizumab is a high-affinity, humanised, IgG4-κ monoclonal antibody targeting PD-1 that was first approved by the FDA in 2017 according to the results of the phase Ib KEYNOTE 012 cohort amplification trial.^64^ In 2019, a phase III clinical study using pembrolizumab (KEYNOTE 048) in the treatment of relapsed or metastatic HNSCC reported superior treatment results from pembrolizumab. In the interim analysis, pembrolizumab combined with chemotherapy improved the OS in the general population compared with cetuximab combined with chemotherapy (13.0 months vs 10.7 months, *P* = 0.003 4). Based on the observed efficacy and safety results, pembrolizumab and chemotherapy are now the first-line treatment for patients with recurrent or metastatic HNSCC, whereas pembrolizumab monotherapy is the first-line treatment for patients with relapsed or metastatic PD-L1-positive HNSCC.^65^

Clinical trials have shown conflicting results. There are clinical responses in patients with PD-L1^−^ cancer, whereas some patients with PD-L1 expression are resistant to immunotherapy. Therefore, the relationship of PD-L1 status with disease outcomes and the appropriate cut-off for PD-L1 expression need to be determined. PD-L1 immunohistochemical staining results are not always reliable enough to accurately predict the response to immunotherapy, and other biomarkers may be required. Qualitative and quantitative multiparametric analyses are helpful. Although the expression of PD-L1 is a main predictor, it must be balanced with other markers, such as the density, composition and activation state of inflammatory cells in the tumour microenvironment.^66^ The combination of the expression of PD-L1 in HNSCC tumour cells and the presence of CTLs is more predictive of tumour cell response than single expression in tumour cells.^67^

Both nivolumab and pembrolizumab are monoclonal antibodies that block PD-1. Durvalumab (Imfinzi; Medimmune/AstraZeneca) is another fully human monoclonal antibody that blocks the binding of PD-L1 to the receptors PD-1 and CD80. In the HAWK phase II study, it was used in patients with recurrent and/or metastatic HNSCC in whom PD-L1 expression was found in more than 25% of tumour cells after the failure of platinum-based chemotherapy.^68^

At the same time, another ligand, PD-L2, is receiving more attention. Researchers evaluated PD-L2 expression in tumour tissue using immunohistochemistry. In addition, the relationship between clinical response and PD-L2 status in human tumour tissues from patients with recurrent or metastatic HNSCC treated with pembrolizumab was also evaluated. The results showed that the levels of PD-L2 and PD-L1 in the tumour had a very significant relationship (*P* < 0.000 1).^69^ PD-L2 expression can also be detected in tumours with no PD-L1 expression. This indicates the predictive power of PD-L2 is independent of PD-L1.^70^ Patients with PD-L1^+^ and PD-L2^+^ tumours (27.5%) had a stronger response than those with only PD-L1^+^ tumours (11.4%). Patients with PD-L2^+^ tumours have a longer median PFS and longer overall survival than patients with PD-L2^-^ tumours.

Patients with tumour cells with simultaneous high levels of PD-1 in TILs and PD-L1 may have shorter disease-free survival than patients with single expression of either marker.^71^ These data highlight that PD-1/PD-L1 overexpression in cancer could have adverse outcomes.^72^ The microenvironment of HNSCC requires further exploration and understanding using a multiparametric approach. Some ongoing clinical trials are shown in supplementary Table 1.

### CTLA-4

Since CTLA-4 can bind to B7, it prevents the interaction between B7 and the co-stimulatory molecule CD28 and restricts the proliferation of T cells and the production of IL-2.^73^ Blocking CTLA-4 can abolish the inhibition of T cells, leading to an antitumour immune response in the host. There are currently two human anti-CTLA-4 antibodies used in phase III clinical trials, ipilimumab and tremelimumab. Phase I/II studies have shown that both antibodies are safe and show some activity as monotherapy or when combined with IL-2 or conventional chemotherapy.^74,75^

In non-small-cell lung carcinoma or small-cell lung carcinoma, a large phase II clinical study of patients found that ipilimumab increased overall PFS and immune-related PFS. This indicated the unique response characteristics of ipilimumab, but the OS did not increase.^76^ In addition, patients receiving ipilimumab after chemotherapy had the greatest increase in PFS, indicating that ipilimumab was most effective after chemotherapy-induced tumour antigen release.^77^ Tremelimumab could be used as second-line or third-line treatment in patients with relapsed malignant mesothelioma. These data suggest that ipilimumab may be promising for HNSCC, but researchers should be aware of the unique properties of the drug when considering treating patients with the therapy. More clinical trials are being used in combination with other drugs or treatments (nivolumab, relatlimab or cetuximab; NCT04080804).

### GITR

AMG 228 is an agonistic human IgG1 monoclonal antibody that binds to human GITR. In one study, the investigators selected 30 patients (over 18 years old) with recurrent malignant solid tumours who met the experimental requirements (NCT02437916), including patients with non-small-cell lung carcinoma, HNSCC, melanoma and colon adenocarcinoma. AMG 228 was administered to patients intravenously every three weeks and was divided into two phase escalation doses: a single patient cohort (3, 9, 30, and 90 mg) followed by a “rolling 6” design (*n* = 2–6; 180, 360, 600, 900 and 1 200 mg). Finally, drug safety, pharmacokinetics, dose-limiting toxicity (DLT), pharmacodynamics, and maximum tolerated dose (MTD) were assessed based on the patients’ response. The results showed that the drug was well tolerated. Patients did not show DLT, and the maximum administered dose did not reach the MTD. There was no evidence of T cell activation or antitumour activity with a single application of AMG 228 therapy.^78^

### TIM-3

Kim et al. found that TIM-3 blockade alone cannot significantly improve the overall survival rate of in vitro models, nor can it benefit the effector cell population. However, it increases the expression of inflammatory cytokines such as IFN-γ, TNF-α and IL17α in tumours. In addition, they identified that TIM-3 inhibition can increase CD4^+^ T cell dependence, as CD4^+^ T cell depletion eliminates the anticancer effect of TIM-3 inhibition.^79^ Clinical trials are currently evaluating the role of combined blockade of TIM-3 and PD-1/PD-L1 in various advanced solid tumours (NCT02817633).

### LAG-3

In a phase I/IIa study, the anti-LAG-3 antibody BMS-986016 was used in combination with nivolumab (NCT01968109) in patients with relapsed melanoma treated with anti-PD-1 antibodies. In this trial, patients with melanoma must have had prior anti-PD-1/PD-L1 antibody (± anti-CTLA-4 antibodies or BRAF/MEK inhibitors) treatment and progressive disease. These patients (*n* = 43) received BMS-986016 (80 mg) and nivolumab (240 mg). The results showed that 70% of these patients (*n* = 30) had prior anti-CTLA-4 antibody therapy, 47% (*n* = 20) had ≥3 prior therapies, and 35% (*n* = 15) had *proto-oncogene B-Raf and v-Raf murine sarcoma viral oncogene homologue B* (*BRAF*) mutations. Of the 31 patients who were effectively evaluated, the objective response rate (OCR) was 16%, and the disease control rate (DCR) was 45%, with efficacy observed even in some patients who did not respond to previous anti-PD-1 therapy.^80^

### TIGIT

TIGIT-targeting therapy is still in the early stages of clinical development. The antagonistic anti-TIGIT antibody OMP-313M32 (NCT03119428) was evaluated as a single drug, and two antibodies were combined with PD-1 blockers to evaluate MTIG7192A (NCT02794571) and BMS-986207 in some advanced primary brain tumours or other primary tumours (NCT02913313).

### VISTA

Among tumour-infiltrating T cells and M2 macrophages (those macrophages that decrease inflammation and encourage tissue repair) in patients with localized and metastatic prostate cancer treated with ipilimumab, VISTA, PD-1 and PD-L1 have been shown to be co-upregulated. To date, there has been one terminated clinical trial utilizing an anti-VISTA monoclonal antibody (JNJ-61610588) in advanced cancers (NCT02671955).

## Combination immune checkpoint inhibition

Inhibitors of PD-1/PD-L1 have been shown to be effective in the treatment of many cancer types. However, it has been recently reported that in the process of inhibiting the PD-1/PD-L1 axis, the expression of other immune checkpoints, such as TIM-3, is increased, which may be related to adaptive resistance.^81^ Combined checkpoint inhibitors (CPIs) have been successfully used to improve tumour immune response and survival, and an increasing number of checkpoint inhibitors are targeting many costimulatory and cosuppressive interactions.

### PD-1/CTLA4

In a clinical case report, for the first time, the combination of the anti-PD-1 antibody nivolumab (3 mg·kg^−1^ body weight every 2 weeks) and the anti-CTLA4 antibody ipilimumab (1 mg·kg^−1^ body weight every 6 weeks) was administered to a patient with HNSCC.^82^ After 3 weeks of treatment with the drugs, computerized tomography (CT) examination of the patient showed a cancer response 4 months after surgery. However, magnetic resonance imaging (MRI) showed local recurrence after 7 months. The expression of PD-L1 and CTLA-4 significantly decreased. There was no significant change in PD-L2 expression or the numbers of B cells, T cells, Th cells, cytotoxic and regulatory T lymphocytes, or NK cells. There are some ongoing clinical trials of the combination of PD-1 and CTLA-4 inhibitors in HNSCC (supplementary Table 2).

### PD-1/GITR

Clinical data have shown that anti-GITR and anti-PD-1 antibody combination therapy can enhance the antitumour activity of T cells.^83^ BMS-986156 is a agonistic human IgG1 monoclonal antibody. It binds with GITR, promoting effector T cell activation, and may reduce or inactivate Treg cells. There has been a clinical phase I/IIa study testing the role of BMS-986156 ± nivolumab (anti-PD-1 mAb) in malignant solid tumours (NCT02598960).

In a dose-escalation study, 66 patients were asked to receive BMS-986156 (10–800 mg) or BMS-986156 (30–800 mg) with nivolumab (240 mg) every 2 weeks. The results showed that BMS-986156 ± nivolumab treatment was well tolerated. There was no obvious dose-limiting toxicity. Low immunogenicity and significant antitumour activity were observed with BMS-986156 and nivolumab at doses predicted to be biologically active.^84^

### PD-1/LAG-3

Another phase I/IIa dose-escalation and dose-expansion study, CA224–020, explored use of BMS-986016 (an anti-LAG3 antibody) as monotherapy and in combination with nivolumab for advanced solid tumours, including an HNSCC cohort.

### Combinations with other therapies

#### Radiotherapy

It is estimated that more than 50% of patients with solid tumours receive radiotherapy (RT), which has an advantage over chemotherapy in that it limits systemic toxicity.^85^ In combination with immune checkpoint inhibitors, RT normalizes the tumour vascular system, enhances the expression of leukocyte adhesion molecules on endothelial cells, and results in secretion of CD8^+^ T-attracting chemokines. RT triggers immunogenic cell death by inducing cell surface exposure of calreticulin (CALR) and ATP secretion via autophagy and IFN-I-induced upregulation of MHC-I, HSP70 exposure, TLR3 signal, high-mobility group box 1 (HMGB1) release and IL-1β release.^86^ RT can induce signals to suppress immune activation, promote infiltration of bone marrow-derived suppressor cells and lead to upregulation of PD-L1.^87^ As RT has certain vaccine characteristics, the combined use of RT with immune checkpoint inhibitors can produce synergistic effects. After receiving RT, some patients may show somatic mutations that produce new antigens, which may become targets for stronger immune responses. The simultaneous use of RT and immune checkpoint inhibitors is considered safe, and immune-related adverse events have not been increased significantly. It is worth considering that high-dose radiotherapy may limit effective tumour immunity by reducing the production of IFN-I in irradiated tumours, whereas low-dose radiotherapy may cause the lymphocyte pool to shrink and eventually render immunotherapy ineffective.

#### Chemotherapy

Currently, all tumour-related antibodies are used in combination with chemotherapeutic drugs because they are less effective as monotherapy.^88^ Chemotherapy can assist immune checkpoint inhibitor treatment by releasing neoantigens or reshaping the tumour microenvironment by depleting Tregs and MDSCs. Chemotherapy can assist immunotherapy by downregulating PD-L2 expression on DCs and tumour cells, inducing APCs to mature, restoring tumour visibility, and upregulating MHC-I expression.^89^ Pemetrexed, carboplatin chemotherapy and pembrolizumab are approved therapies. Alterations to T cell-related genes, such as upregulation of the IFN-γ pathway and IFN-I, are involved in recruitment and activation.^90^ Pembrolizumab has no overlapping toxicity with these drugs. A phase II study evaluated the effectiveness of a combination of ipilimumab, paclitaxel and carboplatin in patients with stage IV non-small-cell lung carcinoma (NCT02279732).^77^ In addition, a comparative study of nivolumab combined with ipilimumab as the standard of care (extreme regimen) for first-line treatment of HNSCC is ongoing (NCT02741570). However, chemotherapy may cause lymphopenia and neutropenia, which may interfere with the mechanism of checkpoint inhibitors by inhibiting the clonal expansion of effector lymphocytes. Therefore, chemotherapy should be carefully considered.

#### Combinations with small molecule drugs (BRAF/TKs)

Small molecule drugs are usually signalling inhibitors that can specifically block the signalling pathways necessary for tumour growth and proliferation, thereby achieving the purpose of treatment.

In ~5% of human malignancies, *BRAF* oncogene mutations cause dysregulation, resulting in structural activation of the mitogen-activated protein kinase (MAPK) pathway and activation of mitogen-activated protein kinase (MEK).^91^ The activation of *BRAF* can lead to the expression of anti-inflammatory cytokines and inhibit the function of TILs. The upregulation of PD-L1 is related to the formation of resistance to BRAF inhibitors.^92^ A phase Ib trial demonstrated the use of BRAF and MEK inhibitors (cobimetinib and vemurafenib) in combination with atezolizumab (anti-PD-L1) in patients with metastatic melanoma with the *BRAF V600E* mutation. Triple therapy improved clinical efficacy and extended survival.^93^ In addition, there was a phase I trial comparing the safety and tolerability of durvalumab (MEDI4736) in combination with dabrafenib (BRAF inhibitor) and trametinib (BRAF inhibitor) with those of durvalumab in combination with trametinib (MEK inhibitor) alone (NCT02027961). A clinical trial of ipilimumab with or without dabrafenib, trametinib or nivolumab in patients with metastatic or unresectable melanoma is ongoing (NCT01940809).

Tyrosine kinases (TKs) have vital functions in growth factor signal transduction. Activated TKs can promote tumour cell proliferation, anti-apoptosis mechanisms, angiogenesis and metastasis.^94^ Sunitinib is a cellular signalling inhibitor that targets multiple tyrosine kinase receptors, including platelet-derived growth factors (PDGFRs), vascular endothelial growth factor receptors (VEGFRs) and c-KIT.^95^ A phase III clinical trial showed that pembrolizumab and avelumab in combination with the multi-TK inhibitor axitinib can benefit patients with renal cell carcinoma.^96^ Small molecules targeting c-KIT can reduce immunosuppressive MDSCs and show good activity when combined with anti-PD-1 or anti-CTLA-4 antibodies. The small molecule drug IPI-549 selectively inhibits the PI3K signalling pathway, which is highly expressed on myeloid cells and promotes migration in murine models of breast carcinoma and melanoma.^97^

#### Cancer Vaccines

Cancer vaccines have antigenicity and immunogenicity. For example, DC vaccines induce cancer-specific immune responses by carrying neoantigens encoded in DNA or mRNA or specific cell lysates.^98^ However, cancer vaccines do not combat the suppression of the tumour microenvironment, and studies found that molecules binding to immune checkpoint inhibitors on activated exhausted T cells could improve treatment outcomes. Using dual anti-CTLA-4/anti-PD-1 inhibitors and a DNA vaccine in mouse melanoma could increase the infiltration of CD8^+^ T cells into the tumour.^99^ Currently, several clinical trials evaluating mRNA cancer vaccines are being conducted in combination with immune checkpoint inhibitors (NCT03633110, supplementary Table 2).

## Conclusions

Immunotherapy is a promising approach to the treatment of patients with HNSCC. Both single-drug therapy and combination therapy have been shown to reduce morbidity and prolong the survival of patients with carcinoma. However, compared with conventional chemoradiotherapy, many immunotherapies take longer to achieve a clinical response and may even lead to tumour pseudoprogression. Differences in dose sequence and timing and in drug combinations may affect the magnitude and duration of immune-mediated antitumour activity. Therefore, as the understanding of the process of immune tumour cell death continues to deepen, guidelines will become available for the development of comprehensive treatment methods that enhance antitumour immunity and the sensitivity of tumour tissues to effector cell killing.^100^

However, we are still in the early stages of understanding the potential of immunotherapy and know little about the best way to combine surgery, chemotherapy, and radiotherapy with immunotherapy. Recently, upregulation of PD-L1 has been demonstrated in cancers treated with chemotherapy. This may indicate a potential benefit of the combined use of immunotherapy, chemotherapy and vaccines in the treatment of cancers.^101^

In addition, there are many challenges that need to be overcome to realize the clinical effects of immunotherapy: the choice of patients, the need for predictive biomarkers, and the need to test the relative efficacy of several immunotherapies over traditional drugs. In short, scientists still need to perform more investigations to achieve ideal treatments for clinical use to improve the survival of patients with HNSCC.

## Supplementary information

Table 1

Table 2
