# Supplementary material for: Immune checkpoint pathways in immunotherapy for head and neck squamous cell carcinoma
Source: Int J Oral Sci. 2020 May 28;12:16. doi: 10.1038/s41368-020-0084-8 (PMC7253444; doi:10.1038/s41368-020-0084-8)
Supplement: Supplementary file 1 — Table 1 [file 41368_2020_84_MOESM1_ESM.docx]

| **Table 1 List of current clinical trials on monotherapy utilizing checkpoint inhibitors in head and neck squamous cell carcinoma** | | | | | |
| --- | --- | --- | --- | --- | --- |
|  |  |  |  |  |  |
| Clinical trial | Phase | Immune target | Drug | Trial Title | Results/Status |
| NCT01860430 | I | PD-1 | Ipilimumab | Ipilimumab, Cetuximab, and Intensity-Modulated Radiation Therapy in Treating Patients with Previously Untreated Stage III-IVB Head and Neck Cancer | Ongoing study |
| NCT02105636 | III | PD-1 | Nivolumab | Trial of Nivolumab vs therapy of investigator’s choice in recurrent or metastatic head and neck carcinoma (CheckMate 141) | Ongoing study |
| NCT02207530 | II | PD-L1 | MEDI4736 | Phase II Study of MEDI4736 monotherapy in treatment of recurrent or metastatic squamous cell carcinoma of the head and neck | Ongoing study |
| NCT02274155 | I | OX40 | MEDI6469 | Anti-OX40 Antibody in head and neck cancer patients | Ongoing study |
| NCT02426892 | II | PD-1 | Nivolumab | Nivolumab and HPV-16 vaccination in patients with HPV-16 positive incurable solid tumours | Ongoing study |
| NCT02643550 | I/II | PD-1 | Monalizumab | Study of Monalizumab and Cetuximab in patients with recurrent or metastatic squamous cell carcinoma of the head and neck | Ongoing study |
| NCT03538028 | I | LAG-3 | INCAGN02385 | A Safety and Tolerability Study of INCAGN02385 in Select Advanced Malignancies | Ongoing study |
| NCT02759575 | I/II | PD-1 | Pembrolizumab | A study of chemoradiation plus pembrolizumab for locally advanced laryngeal squamous cell carcinoma | Ongoing study |
| NCT02769520 | II | PD-1 | Pembrolizumab | Efficacy Study of Pembrolizumab in relapsed, locally recurrent squamous cell cancer of the head and neck | Ongoing study |
| NCT02812524 | I | CTLA-4 | Ipilimumab | Ipilimumab for head and neck cancer patients | Ongoing study |
| NCT03652077 | I | TIM-3 | INCAGN02390 | A Safety and Tolerability Study of INCAGN02390 in Select Advanced Malignancies | Ongoing study |
| NCT02827838 | I | PD-L1 | Durvalumab | Durvalumab before surgery in treating patients with oral cavity or oropharynx cancer | Ongoing study |
| NCT02952586 | III | PD-L1 | Avelumab | Study to compare Avelumab in combination with standard of care chemoradiotherapy (SoC CRT) versus SoC CRT for definitive treatment in patients with locally advanced squamous cell carcinoma of the head and neck (JAVELIN HEAD AND NECK 100) | Ongoing study |
| NCT03040999 | III | PD-1 | Pembrolizumab | Study of Pembrolizumab (MK-3475) or Placebo with chemoradiation in participants with locally advanced head and neck squamous cell carcinoma (MK-3475-412/KEYNOTE-412) | Ongoing study |
| NCT03849469 | I | LAG-3 | XmAb^®^22841 | A Study of XmAb®22841 Monotherapy & in Combination w/ Pembrolizumab in Subjects w/ Selected Advanced Solid Tumors (DUET-4) | Ongoing study |
| NCT03628677 | I | TIGIT | AB154 | A Study to Evaluate the Safety and Tolerability of AB154 in Participants with Advanced Malignancies | Ongoing study |
| NCT03799003 | I | GITR | ASP1951 | A Study of ASP1951 in Subjects with Advanced Solid Tumors | Ongoing study |
| NCT04164238 | II | PD-1 | Toripalimab | Neoadjuvant Anti-PD-1 Antibody (Toripalimab) or Combined with Chemotherapy in HNSCC Patients | Ongoing study |
