# Supplementary material for: Immune checkpoint pathways in immunotherapy for head and neck squamous cell carcinoma
Source: Int J Oral Sci. 2020 May 28;12:16. doi: 10.1038/s41368-020-0084-8 (PMC7253444; doi:10.1038/s41368-020-0084-8)
Supplement: Supplementary file 2 — Table 2 [file 41368_2020_84_MOESM2_ESM.docx]

| **Table 2 List of current clinical trials on Combination therapy utilizing checkpoint inhibitors in head and neck squamous cell carcinoma** | | | | | |
| --- | --- | --- | --- | --- | --- |
| Clinical trial | Phase | Immune target | drug | Trial Title | Results/Status |
| NCT02997332 | I | PD-L1 | Durvalumab, Docetaxel, Cisplatin, 5 Fluorouracil | Durvalumab in Combination with Docetaxel, Cisplatin and 5-FU for Locally Advanced Head and Neck Squamous Cell Carcinoma | Ongoing study |
| NCT01935921 | I | CTLA-4 | Ipilimumab, Cetuximab, IMRT | Ipilimumab, Cetuximab, and Intensity-Modulated Radiation Therapy in Treating Patients with Previously Untreated Stage III-IVB Head and Neck Cancer | Ongoing study |
| NCT02764593 | I | PD-1 | Nivolumab, Cisplatin, Cetuximab, IMRT | Safety testing of adding Nivolumab to chemotherapy in patients with intermediate and high-risk local-regionally advanced head and neck cancer | Ongoing study |
| NCT03019003 | Ib/II | PD-L1, CTLA-4 | Durvalumab, Tremelimumab, Azacitidine | Azacitidine, Durvalumab, and Tremelimumab in Recurrent and/or Metastatic Head and Neck Cancer Patients | Ongoing study |
| NCT02521870 | Ib/II | PD-1, TLR9 | Pembrolizumab, SD-101 | A Trial of Intratumoral Injections of SD-101 in combination with Pembrolizumab in patients with metastatic melanoma or recurrent or metastatic head and neck squamous cell carcinoma | Ongoing study |
| NCT02707588 | II | PD-1 | Pembrolizumab, RT/cetuximab, RT | Tolerance and Efficacy of Pembrolizumab or Cetuximab Combined with RT in Patients with Locally Advanced HNSCC (PembroRad) | Ongoing study |
| NCT02289209 | II | PD-1 | MK-3475 (Keytruda, pembrolizumab), Reirradiation | Reirradiation With Pembrolizumab in Locoregional Inoperable Recurrence or Second Primary Squamous Cell CA of the Head and Neck | Ongoing study |
| NCT02454179 | II | PD-1 | Pembrolizumab, Acalabrutinib | Study of the Combination of Acalabrutinib (ACP-196) and Pembrolizumab in Advanced Head and Neck Squamous Cell Carcinoma (KEYNOTE147) | Ongoing study |
| NCT03051906 | II | PD-L1 | Durvalumab, cetuximab, RT | Durvalumab, Cetuximab and Radiotherapy in Head Neck Cancer | Ongoing study |
| NCT02319044 | II | PD-L1, CTLA-4 | Durvalumab, tremelimumab | Phase II Study of MEDI4736, Tremelimumab, and MEDI4736 in combination with Tremelimumab squamous cell carcinoma of the head and neck | Ongoing study |
| NCT02718820 | I/II | PD-1 | Pembrolizumab, Docetaxel | Pembrolizumab Plus Docetaxel for the Treatment of Recurrent or Metastatic Head and Neck Cancer | Ongoing study |
| NCT02499328 | I/II | PD-L1 | Durvalumab, AZD5069 | Study to Assess MEDI4736 With Either AZD9150 or AZD5069 in advanced solid tumours & relapsed metastatic squamous cell carcinoma of head & neck | Ongoing study |
| NCT02369874 | III | PD-L1, CTLA-4 | MEDI4736, Tremelimumab | Study of MEDI4736 monotherapy and in combination with tremelimumab versus standard of care therapy in patients with head and neck cancer | Ongoing study |
| NCT02499328 | I/II | PD-L1, CTLA-4 | Tremelimumab, AZD9150, AZD5069, MEDI4736 | Study to Assess MEDI4736 With Either AZD9150 or AZD5069 in Advanced Solid Tumors & Relapsed Metastatic Squamous Cell Carcinoma of Head & Neck | Ongoing study |
| NCT02291055 | I/II | PD-L1 | Durvalumab, ADXS11-001 | Phase I-II Study of ADXS11-001 or MEDI4736 Alone or Combo in cervical or HPV+ head & neck cancer | Ongoing study |
| NCT01860430 | I | CTLA-4 | Ipilimumab, cetuximab, IMRT | Ipilimumab, Cetuximab, and Intensity-Modulated Radiation Therapy in Treating Patients with Previously Untreated Stage III-IVB Head and Neck Cancer | Ongoing study |
| NCT04080804 | II | PD-1, CTLA-4, LAG-3 | Nivolumab, Relatlimab, Ipilimumab | Study of Safety and Tolerability of Nivolumab Treatment Alone or in Combination with Relatlimab or Ipilimumab in Head and Neck Cancer | Ongoing study |
| NCT03633110 | I/II | PD-1 | GEN-009, Nivolumab, Pembrolizumab | Safety, Tolerability, Immunogenicity, and Antitumor Activity of GEN-009 Adjuvanted Vaccine | Ongoing study |
| NCT03625323 | II | PD-1/LAG-3 | Pembrolizumab, IMP321 | Combination Study with Soluble LAG-3 Fusion Protein IMP321 and Pembrolizumab in Patients With Previously Untreated Unresectable or Metastatic NSCLC, or Recurrent PD-X Refractory NSCLC or With Recurrent or Metastatic HNSCC (TACTI-002) | Ongoing study |
